# Oyster Farming, Temperature, and Plankton Influence the Dynamics of Pathogenic Vibrios in the Thau Lagoon

**DOI:** 10.3389/fmicb.2018.02530

**Published:** 2018-10-24

**Authors:** Carmen Lopez-Joven, Jean-Luc Rolland, Philippe Haffner, Audrey Caro, Cécile Roques, Claire Carré, Marie-Agnès Travers, Eric Abadie, Mohamed Laabir, Delphine Bonnet, Delphine Destoumieux-Garzón

**Affiliations:** ^1^IHPE, Université de Montpellier, CNRS, Ifremer, UPVD, Montpellier, France; ^2^MARBEC, Université de Montpellier, CNRS, Ifremer, IRD, Montpellier, France; ^3^Ifremer, Laboratoire de Génétique et Pathologie des Mollusques Marins, LGPMM-SG2M, La Tremblade, France

**Keywords:** *Vibrio*, shellfish farming, bivalve mollusks, mortality outbreak, phytoplankton, zooplankton

## Abstract

*Vibrio* species have been associated with recurrent mass mortalities of juvenile oysters *Crassostrea gigas* threatening oyster farming worldwide. However, knowledge of the ecology of pathogens in affected oyster farming areas remains scarce. Specifically, there are no data regarding (i) the environmental reservoirs of *Vibrio* populations pathogenic to oysters, (ii) the environmental factors favoring their transmission, and (iii) the influence of oyster farming on the persistence of those pathogens. This knowledge gap limits our capacity to predict and mitigate disease occurrence. To address these issues, we monitored *Vibrio* species potentially pathogenic to *C. gigas* in 2013 and 2014 in the Thau Lagoon, a major oyster farming region in the coastal French Mediterranean. Sampling stations were chosen inside and outside oyster farms. Abundance and composition of phyto-, microzoo-, and mesozooplankton communities were measured monthly. The spatial and temporal dynamics of plankton and *Vibrio* species were compared, and positive correlations between plankton species and vibrios were verified by qPCR on isolated specimens of plankton. *Vibrio crassostreae* was present in the water column over both years, whereas *Vibrio tasmaniensis* was mostly found in 2013 and *Vibrio aestuarianus* was never detected. Moreover, *V. tasmaniensis* and *V. crassostreae* were found both as free-living or plankton-attached vibrios 1 month after spring mortalities of the oyster juveniles. Overall, *V. crassostreae* was associated with temperature and plankton composition, whereas *V. tasmaniensis* correlated with plankton composition only. The abundance of *Vibrio* species in the water column was similar inside and outside oyster farms, suggesting important spatial dispersion of pathogens in surrounding areas. Remarkably, a major increase in *V. tasmaniensis* and *V. crassostreae* was measured in the sediment of oyster farms during cold months. Thus, a winter reservoir of pathogenic vibrios could contribute to their ecology in this Mediterranean shellfish farming ecosystem.

## Introduction

*Vibrio* species are causal agents of epizootics, zoonoses, and epidemics (Austin, 2010; Le Roux et al., 2015). Over the past two decades, strains of *Vibrio aestuarianus* and those of the Splendidus clade have been associated with mortality among farmed oysters (Soletchnik et al., 1999; Gay et al., 2004; Lemire et al., 2015). Whereas *V. aestuarianus* is known as a major pathogen to adult oysters (Travers et al., 2017), strains of the Splendidus clade (*Vibrio tasmaniensis* and *Vibrio crassostreae*) are associated with a multifactorial disease affecting spats and juveniles (Gay et al., 2004; Lemire et al., 2015; Bruto et al., 2017; de Lorgeril et al., 2018), which is triggered by herpes virus OsHV-1 μVar (Segarra et al., 2010; Martenot et al., 2011). This disease, referred to as Pacific oyster mortality syndrome, occurs seasonally when seawater temperature reaches 16–24°C (Pernet et al., 2012, 2014). Mortality is observed in the summer season in oyster farms on the French Atlantic coast, and in spring and autumn along the Mediterranean coast, which is characterized by warmer seawater temperature.

The highly diverse Splendidus clade is ubiquitous in marine coastal environments (Pérez-Cataluña et al., 2016). It encompasses various *Vibrio* species, some of which are pathogenic to oysters (Lemire et al., 2015; Bruto et al., 2017). It is now accepted that detection of the Splendidus clade, which has been widely used in environmental surveys (Pernet et al., 2012; Vezzulli et al., 2015), cannot be considered indicative of pathogen prevalence. Currently, risk assessment for *Vibrio* species pathogenic to humans is based largely on detection of virulence factors (Food and Agriculture Organization of the United Nations/World Health Organization [FAO/WHO], 2011), some of which have been identified also in populations of the Splendidus clade. For example, the major porin (OmpU) of *V. tasmaniensis* LGP32, was shown to be required for hemocyte invasion and subsequent cytolysis and virulence expression (Duperthuy et al., 2011; Vanhove et al., 2015, 2016). In *V. crassostreae*, whose known members are pathogenic to oysters, virulence is dependent on both a genomic region referred to as R5 (Lemire et al., 2015) and a virulence plasmid (Bruto et al., 2017). The recent discovery of virulence factors in *V. tasmaniensis* and *V. crassostreae* has enabled a more accurate monitoring of Splendidus clade populations potentially pathogenic to oysters.

Contrary to the Splendidus clade, *V. aestuarianus* causes an almost clonal disease. Strains of *V. aestuarianus* isolated during adult oyster mortalities are mostly pathogenic and form phylogenetically coherent virulent lineages, in which only a few strains have lost their pathogenicity. The loss of virulence in those rare cases has been attributed to a frame shift in a regulatory gene controlling the expression of the metalloprotease *Vam* (Goudenège et al., 2015). As a consequence of clonality, *dnaJ* detection is considered a reliable way of identifying the presence of *V. aestuarianus* (Saulnier et al., 2009) in the environment.

While mortalities of juvenile oysters have been documented repeatedly over the past years, causing dramatic losses to French oyster production^1^, environmental drivers of the disease’s recurrence have been poorly investigated. To our knowledge, the only environmental survey exploring the spatial and temporal distribution of *Vibrio* species of the Splendidus clade and *V. aestuarianus* in Mediterranean marine coastal systems has been performed in the Goro Lagoon, Italy (Vezzulli et al., 2015). In this lagoon, which is used for clam but not oyster farming, the authors found the Splendidus clade associated with mollusks (oysters and mussels), plankton, sediment, and seawater in both winter and summer. *V. aestuarianus* was found in summer and at very low levels in the sediment in winter. To predict and mitigate disease occurrence, it is paramount to understand the recurrence of the Pacific oyster mortality syndrome in marine ecosystems exploited for oyster farming. This requires gaining more insight on the environmental reservoirs of vibrios associated with the disease and environmental factors driving their dynamics and transmission to oysters.

The present study aimed to investigate the seasonal dynamics of vibrios potentially pathogenic to oysters in the Thau Lagoon, a Mediterranean marine ecosystem with a significant oyster farming activity (20% of the total surface). This lagoon has seen repeated dramatic mortalities of oyster juveniles since 2008 (Pernet et al., 2012), causing important economic losses. A monthly monitoring of *Vibrio* species was carried out over 2 years (2013–2014). Environmental parameters of the water column were monitored monthly to identify potential relationships between the abundance of vibrios and phyto-, microzoo-, and mesozooplankton, temperature, and salinity. The potential impact of shellfish farming on pathogen dynamics, Vibrio and plankton communities was investigated in the water column both inside and outside oyster farms. Vibrios were monitored in the sediment at both sampling sites during all four seasons. Findings show that (i) the ecology of *V. crassostreae* and *V. tasmaniensis* is influenced by temperature and/or plankton composition, respectively; and (ii) the sediment of oyster farms constitutes a winter reservoir for pathogenic vibrios.

## Materials and Methods

### Study Area and Measurement of Environmental Parameters

**FIGURE 1:**
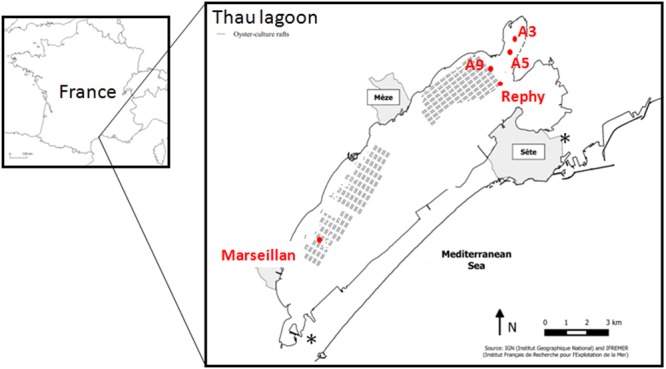
Location and map of the Thau Lagoon. The sampling sites (red dots) are located inside (A9 and Rephy) and outside (A3 and A5) the oyster farms. The position of oyster-culture rafts within farms is indicated by gray blocks. The position of seawater channels is indicated by an ^∗^.

The Thau Lagoon is located in the Languedoc-Roussillon region in the south of France, along the Mediterranean coast (Figure 1). It encompasses a large and shallow marine water body (75 km^2^; depth <10 m; mean depth 4.5 m) connected to the sea by two narrow channels. This lagoon is the most important French shellfish farming site along the Mediterranean coast. Temperature and salinity were measured weekly by the REPHY Ifremer network of stations^2^ at four sites. Two of them, Rephy (N 43°26.058′ and E 003°39.878′) and A9 (N 43°26.340′ and E 003°39.722′), were located inside oyster farms and two, A5 (N 43°26.832′ and E 003°40.232′) and A3 (N 43°27.222′ and E 003°40.461′), were located outside oyster farms (Figure 1). The present work also benefited from (i) the monitoring of spring mortalities of oyster spats in the Thau Lagoon by the Ifremer RescoII network in 2013 and 2014 (Réseau d’Observations Conchylicoles [RESCOII], 2014, 2015) and (ii) the 2014 declarations of juvenile oyster mortalities made by oyster farmers to the Cepralmar sentinel survey (Centre d’Étude Pour la Promotion des Activités Lagunaires et Maritimes [CEPRALMAR], 2015).

### Collection and Size-Fractionation of Seawater Samples

Seawater was sampled from the Thau Lagoon monthly from January 2013 to November 2014. Seawater was collected from the sub-surface (-50 cm) using a pump. Sampling sites were located (i) under an oyster table at the Rephy and A9 sites, and (ii) 1.5–2 km away from oyster tables at the A3 and A5 sites (Figure 1). Temperature and salinity were monitored at each sampling site.

#### Quantitative Polymerase Chain Reaction (qPCR) Monitoring of Vibrios

Seawater was fractionated into two size classes as follows. In one case, 20 L of seawater were filtered on the boat through a 180-μm pore-size nylon membrane. Then, in the laboratory, 500 mL of the filtrate were passed through a 5-μm pore-size MF-Millipore membrane (Nucleopore PC 47 mm), and 50 mL of the resulting liquid were filtered through an additional 0.2-μm pore-size MF-Millipore membrane that was kept for further DNA extraction. This membrane contained free-living vibrios sized 0.2–5 μm. In the other case, 20 L of seawater were directly concentrated through a 20-μm pore-size nylon filter to a final volume of 50 mL. Once in the laboratory, 0.5 mL of the concentrate were deposited onto a 10-μm pore-size MF-Millipore membrane. This membrane contained vibrios associated to plankton particles with size >20 μm.

#### Mesozooplankton Composition Analysis

Briefly, 10 m^3^ of seawater collected between A9 and Rephy (inside oyster tables) and between A3 and A5 (outside oyster tables) were directly concentrated to a final volume of 200 mL using a plankton net with a 63-μm mesh size. The collected zooplankton was fixed immediately in 4% formaldehyde and stored at room temperature until species identification was performed by microscopy. This fraction contained organisms >63 μm in size.

#### Microzooplankton Composition Analysis

Ten liters of seawater, collected inside (Rephy) and outside (A5) oyster farms, were size-fractionated on the boat with a 180-μm pore-size nylon filter and 10 mL of the filtrate were preserved in 2% Lugol solution. In the laboratory, samples were stored at 4°C until species identification was performed by microscopy. This fraction contained organisms <180 μm in size.

#### Phytoplankton Composition Analysis

Twenty liters of seawater collected in Rephy and A5 were directly concentrated on a 20-μm pore-size nylon filter to a final volume of 50 mL, 10 mL of concentrate were preserved in 2% Lugol solution and stored at 4°C until species identification was performed by microscopy. This fraction contained organisms >20 μm in size.

### Sampling of Surface Sediment and Bacterial Amplification

Core sediment samples were collected in triplicates at different times (spring, summer, autumn 2014, winter and spring 2015) at A5 and Rephy. Samples were then processed for enumeration of culturable vibrios using the most probable number/PCR method (MPN-PCR) (Machado and Bordalo, 2016); results were expressed as colony-forming units (CFUs)/g sediment. Basically, surface sediment of each core (0–1 cm) was used for the enrichment in Alcaline Peptone Water (APW) broth at pH 8.2. For each sediment core, 10 to 10^-4^ g (1:10 series) of sediment was inoculated in 10 mL of APW; the larger amount (10 g) was inoculated in 100 mL of APW. Incubation was performed at 20°C during 24 h. After that, 1 mL of each APW culture was collected for extraction of bacterial DNA and further qPCR detection of *V. aestuarianus, V. tasmaniensis*, and *V. crassostreae* as described below.

### DNA Extraction and qPCR Monitoring of Vibrios

DNA from water column fractions (>20 and 0.2–5 μm) was extracted using the Macherey Nagel Nucleospin tissue kit. DNA was resuspended in 100 μL of water and stored at -20°C. For sediment samples cultured in APW, DNA was extracted from 1 mL of culture medium using the Wizard Genomic DNA Purification kit (Promega). Quantitative real-time PCR was used to monitor vibrio abundance using primer pairs designed to amplify single-copy genes related to virulence in *V. tasmaniensis* (*ompU*), *V. crassostreae* (*R5-2*), or *V. aestuarianus* (*dnaJ*) (Table 1). Vibrio-specific primers (*16S rRNA*) were used for total vibrios (Table 1). qPCR reactions were performed on a LightCycler 480 (Roche Diagnostics). Typically, reactions contained 1 μL of template DNA (at concentrations of 1–40 μg mL^-1^), 1 μL of each primer (3.33 μM), and 3 μL of reaction mixture (SYBR Green Master Mix) in a total volume of 6 μL. Reaction parameters were as follows: 5 min at 95°C (initial denaturation) and 40 cycles of 10 s at 95°C (denaturation); 10 s at 65°C (*R5-2*), 62°C (*ompU* and *16S rRNA*), or 60°C (*dnaJ*) (hybridization); and 10 s at 72°C (elongation). Melting curve profiles were generated by increasing the temperature from 65 to 95 at 0.5°C per second. Amplification products were analyzed using LightCycler software (Roche Diagnostics).

**Table 1:** Primers sequence used for qPCR amplification.

Species	Gene	Primer sequence 5′→3′	Reference
*V. tasmaniensis*	ompU	GTCCTACAGACAGCGATAGC	This study
		GTGGTAAGCCATGATATCGG	
*V. crassostreae*	R5-2	GGATGGGACCAACTACGGTG	This study
		CGTAGCCCGGAGGAAGAATC	
*V. aestuarianus*	dnaJ	GTATGAAATTTTAACTGACCCACAA	Saulnier et al., 2009
		CAATTTCTTTCGAACAACCAC	
All *Vibrio* spp.	16S rRNA	CGGTGAAATGCGTAGAGAT	Tarr et al., 2007
		TTACTAGCGATTCCGAGTTC	

Quantification of microorganisms was achieved by constructing calibration curves using DNA isolated from reference strains (*V. tasmaniensis* LGP32, *V. crassostreae* J2-9, and *V. aestuarianus* Ifremer LPI 02/41) (Supplementary Figure S1). To produce standard curves, the log value of the vibrio DNA concentration was plotted against the crossing point (Cp) value as suggested by the LightCycler 480 2008 Operator’s Manual (Supplementary Figure S1). Primer specificity and efficiency were verified on DNA extracted from a non-exhaustive list from 21 taxonomically diverse *Vibrio* strains (Supplementary Table S1). The number of bacterial cells per liter of seawater was inferred from the number of amplified copies of single-copy genes. The relationship between DNA concentration and the number of bacterial genomes was calculated using the genome size of reference strains used for standard curves (4.98 Mbp for *V. tasmaniensis* LGP32, 5.79 Mbp for *V. crassostreae* J2-9, and 4.19 Mbp for *V. aestuarianus* 02_041).

### *In situ* Assessment of Plankton/Vibrio Associations

Candidate plankton species identified as co-occurring with vibrios by statistical analysis (i.e., plankton species showing statistically significant interspecies relationships with pathogenic vibrios detected in the >20 μm fraction) were manually isolated from formalin-fixed samples of mesozooplankton collected during our *in situ* sampling. DNA was extracted from specimens (pools of individuals belonging to the same taxa) at chosen dates before qPCR was used to determine the presence of vibrios (see above). Negative controls included DNA extracted from a specimen of mesozooplankton present in the same samples but belonging to taxa which did not show any significant correlation with vibrio abundance.

### Statistical Analysis

To investigate the relationship between vibrios (*16S rRNA*) abundance and environmental variables, principal component analysis was conducted on environmental data for each year. Prior to the analysis, data were standardized to 0 mean and unit variance. The two principal components were retained to assess the potential drivers of *Vibrio* spp. abundance. In addition, to elucidating the relative importance of environmental variables for determining *Vibrio* spp. total abundance, we used the data to construct a general linear model. *Vibrio* spp. detected in the >20 μm fraction and in the 0.2–5 μm fraction were analyzed separately. Data were analyzed per year (A5 and Rephy stations being considered together). Predictors tested in the total *Vibrio* spp. abundance model were those known to be important for *Vibrio* spp. dynamics (temperature, salinity) or those that were indicative of plankton input (total phytoplankton and zooplankton abundances, total ciliates and flagellates abundances). Data were normalized and log-transformed.

To investigate interspecies relationships between pathogenic vibrios and plankton, only *Vibrio* species detected in the >20 μm fraction were considered. A5 and Rephy stations were analyzed jointly. Plankton species with low detection (i.e., fewer than three times in a year) were removed from the analysis. Data were normalized and log-transformed.

Statistical analyses were performed on Statistica (v12.0) software. For statistical analysis between vibrio concentrations in the sediment, temperature, and salinity, parametric correlations were calculated using XLStat-Base software.

## Results

### Spring and Autumn Mortalities of Juvenile Oysters in the Thau Lagoon

Four sites located inside (Rephy and A9) and outside (A5 and A3) oyster farms in the Thau Lagoon, characterized by mass mortality events of oyster spats and juveniles, were surveyed over two successive years (2013–2014). Both years showed similar trends in seawater temperature and salinity (Supplementary Figure S2). In 2013, two mortality risk periods of 2 months each were predicted according to Pernet et al. (2012) in May–June and September–October, when seawater was in the 16–24°C range (Supplementary Figure S2). In 2014, the risk period for mortalities expanded from April to October, and it only excluded August. Mortalities of oyster spats occurred in the predicted risk periods as revealed both by the Ifremer RescoII monitoring network and declarations of shellfish farmers to Cepralmar (Supplementary Figure S2). Oyster mortalities could not be monitored in autumn 2013, as only sentinel animals having survived the spring mortality (i.e., individuals resistant to the disease) were maintained in the lagoon by RescoII (Pernet et al., 2012).

### Distinct Dynamics of Vibrios Potentially Pathogenic to Oysters in the Water Column

**FIGURE 2:**
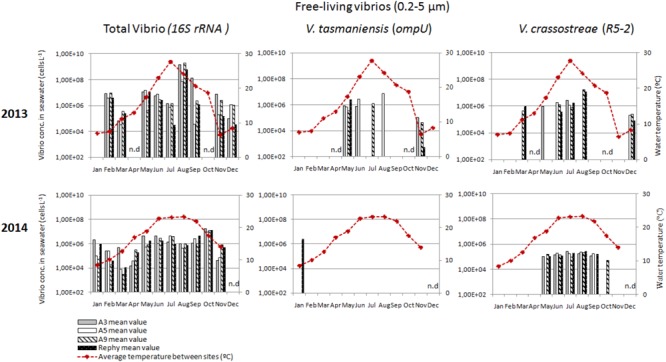
Monthly abundance of free-living vibrios in the water column. Histograms show apparent vibrio concentrations based on qPCR amplification (*16S rRNA, ompU*, and *R5-2*) of DNA extracted from the 0.2–5 μm fraction of the water column. Samples were collected outside the oyster culture area (A3, *gray bars* and A5, *white bars*) and inside an oyster farm (A9, *hatched white bars* and Rephy, *hatched black bars*). Mean seawater temperature (°C) is represented by a red line. n.d., not determined.

**FIGURE 3:**
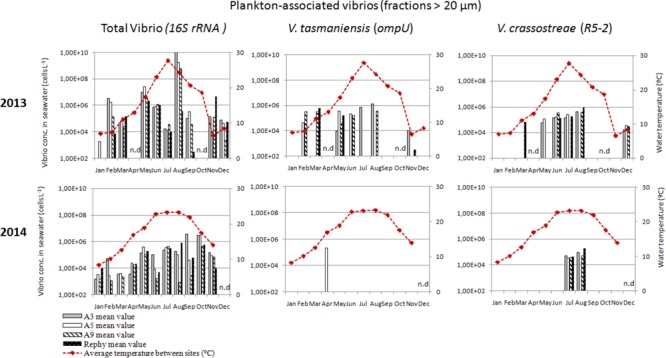
Monthly abundance of plankton-associated vibrios in the water column. Histograms show apparent vibrio concentrations based on qPCR amplification (*16S rRNA, ompU*, and *R5-2*) of DNA extracted from the >20 μm fraction of the water column. Samples were collected outside the oyster culture area (A3, *gray bars* and A5, *white bars*) and inside an oyster farm (A9, *hatched white bars* and Rephy, *hatched black bars*). Mean seawater temperature (°C) is represented by a red line. n.d., not determined.

To monitor the annual dynamics of vibrios in the water column, seawater samples were collected monthly, both inside (Rephy and A9) and outside oyster farms (A5 and A3) over the 2 years. All samples were positive for total vibrios (*16S rRNA*), indicating a high prevalence of vibrios in the Thau Lagoon, both as free-living (0.2–5 μm) and plankton-associated (>20 μm) bacteria (Figures 2, 3). Overall, total vibrio abundance in the water column was poorly dependent on sampling site, but it was two orders of magnitude lower in 2014 than in 2013 (Figures 2, 3).

Vibrios potentially pathogenic for oysters followed distinct environmental dynamics in the Thau lagoon based on apparent concentrations determined by qPCR. *V. crassostreae* (*R5-2*) was recurrent in the water column in 2013–2014; during the warm months it was found both as free-living (3.8 × 10^5^–1.1 × 10^7^ cells L^-1^) and as plankton-associated vibrios (1.3–8.9 × 10^5^ cells L^-1^) (Figures 2, 3). Overall, *V. crassostreae* were ∼100-fold more abundant in 2013 than in 2014 (Figures 2, 3). *V. tasmaniensis* (*ompU*) was found almost exclusively in 2013: free-living *V. tasmaniensis* were detected from spring to autumn (preferentially in May, June, and November, at 2.4 × 10^6^–1.2 × 10^8^ cells L^-1^; Figure 2) whereas plankton-associated *V. tasmaniensis* were detected most of the year at 4.4 × 10^4^–6.3 × 10^5^ cells L^-1^ (Figure 3). Overall, detection of free-living *V. crassostreae* and/or *V. tasmaniensis* in the water column started mainly in spring (May–June), 1 month after the beginning of spring mortalities (Figure 2). *V. aestuarianus* (*dnaJ*) could not be detected during the 2 years surveyed (data not shown).

### Contrasting Annual and Spatial Dynamics of Phyto- and Zooplankton in the Thau Lagoon

**FIGURE 4:**
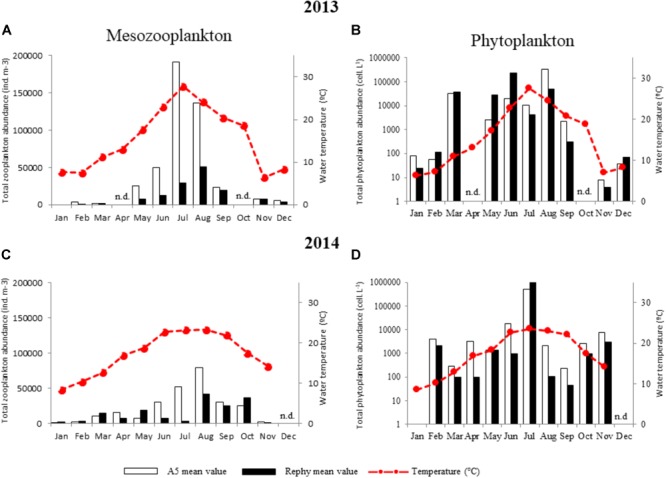
Monthly abundance of total mesozooplankton and phytoplankton. **(A,B)** 2013 and **(C,D)** 2014 monthly values of plankton abundance outside (A5, *white bars*) and inside oyster farms (Rephy, *black bars*). Total mesozooplankton counts (individuals m^-3^) are shown in **(A,C)**. Total phytoplankton counts (cells L^-1^) are shown in **(B,D)**. n.d., not determined.

To identify environmental interactions between potentially pathogenic vibrios to oysters and plankton species that could drive their dynamics in the water column, we determined the monthly abundance of plankton taxa in the water column over the 2 years at both sampling stations (Figure 4). First, results founded with mesozooplankton (>63 μm) species followed a similar trend over the 2 years, with the highest abundance being observed during warm months. Total abundance was up to four times higher in 2013 than in 2014 outside the oysters farm (A5) (Figures 4A,C). Although mesozooplankton abundance was remarkably (up to 20 times) higher outside (A5) than inside oyster farms (Rephy), overall composition of mesozooplankton (27 taxa) did not vary substantially between sites: mesozooplankton species, copepods’ nauplii, and *Oithona* sp. (a copepod) were prevalent at all sites. A large proportion of bivalve larvae were also observed, especially in February–March 2013 (Supplementary Figure S3). The main differences between sites were observed in March 2013 with a dominance of *Polychaeta* outside oyster farms as opposed to bivalves and copepods inside the farms. Overall, 2014 differed from 2013 mostly as a result of a highest contribution of copepods of the *Acartia* and *Paracartia* genera to total mesozooplankton abundance (Supplementary Figure S3).

Microzooplankton (<180 μm) composed of ciliates (*n* = 23 taxa) and tintinnids (*n* = 20 taxa) were collected in the water column only in 2014 (no sampling was performed in 2013). Ciliates were present at both sites with similar dynamics, with *Lee gardiella* sp. and *Mesodinium rubrum* being the most commonly identified taxa (Supplementary Figure S4). Tintinnids presented contrasting dynamics between the two sites, especially in autumn and winter (Supplementary Figure S4). The main differences were observed for *Helicostomella subulata, Tintinnopsis* sp., and *Salpingella* sp. (Supplementary Figure S4).

Phytoplankton (>20 μm) abundance was approximately five times higher in 2013 than in 2014 (Figures 4B,D). Moreover, highly contrasting phytoplankton compositions were observed between years, as determined through taxonomic identification of 47 taxa (Supplementary Figure S5). In 2013, the most commonly found taxa were the diatom *Skeletonema costatum* and the dinoflagellates *Gymnodinium* sp., *Gonyaulax* sp., and *Scrippsiella trochoidea*; whereas 2014 was dominated by the diatom *Chaetoceros* sp. and the dinoflagellates *Protoperidinium* sp. and *Alexandrium* sp. (Supplementary Figure S5). Phytoplankton composition also varied according to sampling site in 2013 (Supplementary Figure S5).

### Plankton Communities Play a Major Role in the Dynamics of Total Vibrios in the Thau Lagoon

**FIGURE 5:**
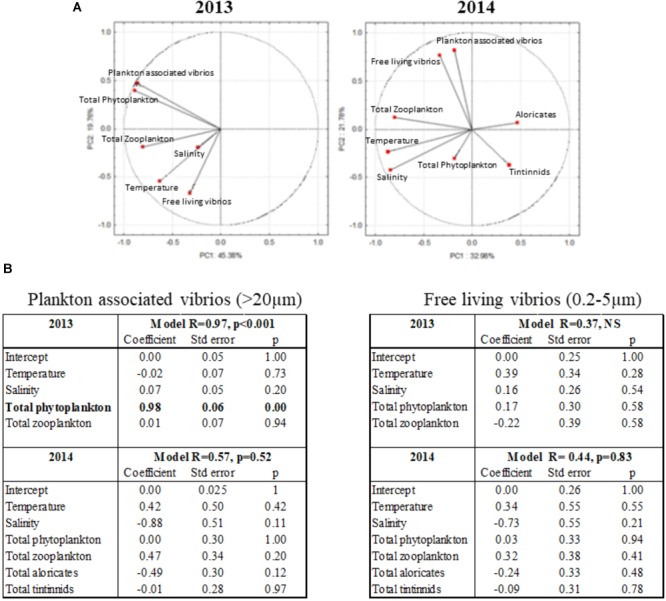
Relationships between total vibrios abundance and environmental parameters. **(A)** Principal component analysis of environmental data from 2013 to 2014: Temperature, Salinity, Total Zooplankton, Total Phytoplankton, Plankton-associated vibrios (>20 μm fraction), Free-living vibrios (0.2–5 μm fraction). For 2014, ciliates data (microzooplankton) are also presented divided into two types of organisms: Aloricates and Tintinnids. **(B)** Results of a general linear model showing the relative importance of environmental variables on the plankton associated vibrios abundance (16S rRNA in the >20 μm fraction) and the free-living vibrios (0.2–5 μm fraction). Results in boldface indicate a significant (*p* < 0.05) effect of the variable.

Correlations between total vibrios (*16S rRNA*), abundance of phyto- and zooplankton, temperature, and salinity were analyzed using data acquired over the 2013–2014 survey. None of these parameters contributed significantly to the annual dynamics of free-living vibrios (Figures 5A,B). Instead, they influenced significantly vibrio associations with plankton species, reflecting notable differences in environmental conditions between the 2 years surveyed (Figure 5A). In 2013, total phytoplankton was the only variable explaining vibrio abundance in the fraction >20 μm (plankton-associated vibrios), as indicated by the general linear model (Figure 5B). In contrast, in 2014, the abundance of plankton-associated vibrios was fully uncoupled from phytoplankton, with no significant correlation being determined with other contributing parameters such as zooplankton and temperature (Figures 5A,B). These data suggest that some plankton species, which do not occur annually, play an important role in driving the association of vibrios with plankton. In agreement with our results on total phytoplankton, most of the significant positive correlations (*p* < 0.05) between phytoplankton species and plankton-associated vibrios (*16S rRNA*) were evidenced in 2013. Thus, in 2013, plankton-associated vibrios correlated with the abundance of five species: the dinoflagellates *Gonyaulax* sp., *Gonyaulax spinifera*, and *Protoperidinium* sp., and the diatoms *Tripos furca* and *Pseudonitzschia* sp. (*r* = 0.568, *r* = 0.665, *r* = 0.685, *r* = 0.516, and *r* = 0.596, respectively; Table 2). Interestingly, in 2014, which differed from 2013 by a very distinct phytoplankton composition, significant positive correlations were observed only with the diatom *Pseudonitzschia* sp. (*r* = 0.493; Table 2). Consistent with an important role of phytoplankton in 2013 only, phytoplankton taxa showing significant positive correlations with vibrios abundance in 2013 were much less prevalent in 2014.

**Table 2:** Correlations between vibrio and plankton species in the water column.

	2013	2014
*V. crassostreae* (R5-2)	*Pseudonitzschia* sp., *r* = 0.503	
	*Gonyaulax spinifera, r* = 0.487	
	*Scrippsiella trochoidea, r* = 0.558	
	*Centropages* sp., *r* = 0.733	
	*Euterpina* sp., *r* = 0.542	
	*Oikopleura dioica, r* = 0.482	
*V. tasmaniensis* (*ompU*)	*Chaetoceros* sp., *r* = 0.601	
	*Obelia* sp., *r* = 0.697	
*All Vibrio* spp. (*16S rRNA*)	*Pseudonitzschia* sp., *r* = 0.596	*Pseudonitzschia* sp., *r* = 0.493
	*Gonyaulax* sp., *r* = 0.568	*Tiarina fusus, r* = 0.472
	*Gonyaulax spinifera, r* = 0.665	*Pseudocalanus* sp., *r* = 0.540
	*Protoperidinium* sp., *r* = 0.685	*Ascidian larvae, r* = 0.595
	*Tripos furca, r* = 0.516	
	Total phyto, *r* = 0.473	
	*Oncaea venusta, r* = 0.555	

*Correlations (*p-*values < 0.05) between vibrio and phytoplankton, microzooplankton and mesozooplankton species abundances according to size class during both years (2013 and 2014) *in situ* monitoring program. Microzooplankton was monitored in 2014 only.*

Significant positive correlations (*p* < 0.05) between total vibrios and the mesozooplankton community occurred mostly in 2013, which was characterized by higher plankton abundance. Plankton-associated vibrios positively correlated with the copepod *Oncaea venusta* (*r* = 0.555) in 2013. The copepod *Pseudocalanus* sp. and the ascidian larvae present in the water column only in 2014, showed significant positive correlation with plankton-associated vibrios (*r* = 0.540 and *r* = 0.595, respectively; Table 2). Correlations between the abundance of microzooplankton species were analyzed in 2014 only, as no sampling was performed in 2013. A significant positive correlation was observed between plankton-associated vibrios and the ciliate *Tiarina fusus* (*r* = 0.472, *p* < 0.05; Table 2).

### Pathogenic Vibrios Show Contrasting Associations With Plankton Species and Abiotic Parameters

The abundance of pathogenic vibrios in the water column was compared to both biotic and abiotic parameters measured in the Thau Lagoon. Among the two pathogenic populations surveyed, only *V. crassostreae* (*R5-2*) showed a positive correlation with temperature, both in its plankton-associated and free-living states. Specifically, plankton-associated *V. crassostreae* correlated positively with temperature in 2013 (*r* = 0.503, *p* < 0.05). Moreover, free-living *V. crassostreae* strongly correlated with temperature (*r* = 0.849, *p* < 0.05) and salinity (*r* = 0.862, *p* < 0.05) in 2014. In contrast, no significant correlation was found between *V. tasmaniensis* (*ompU*) abundance, temperature, and/or salinity.

#### Associations With Phytoplankton Species

Both pathogenic populations displayed distinct associations with phytoplankton species. Significant positive correlations (*p* < 0.05) were found between plankton-associated *V. tasmaniensis* and the diatom *Chaetoceros* sp. (*r* = 0.601). In contrast, plankton-associated *V. crassostreae* showed positive correlations with the dinoflagellates *G. spinifera* (*r* = 0.487) and *S. trochoidea* (*r* = 0.558), found mostly in 2013 (Supplementary Figure S5), and the diatom *Pseudonitzschia* sp. (*r* = 0.503) (Table 2).

#### Associations With Mesozooplankton Species

Significant positive correlations with mesozooplankton species differed according to *Vibrio* species and were apparent mostly in 2013. Plankton-associated *V. tasmaniensis* correlated with abundance of the cnidarian *Obelia* sp. (*r* = 0.697, *p* < 0.05), whereas plankton-associated *V. crassostreae* correlated with abundance of the copepods *Centropages* sp. (*r* = 0.733, *p* < 0.05) and *Euterpina* sp. (*r* = 0.542, *p* < 0.05), as well as the appendicularian *Oikopleura dioica* (*r* = 0.482, *p* < 0.05) (Table 2). As for phytoplankton, fewer mesozooplankton species correlated with pathogenic *Vibrio* species in 2014 (Supplementary Figure S3).

#### Associations With Microzooplankton Species

Correlations between abundance of microzooplankton species and plankton-associated pathogenic *Vibrio* species were analyzed in 2014 only, as no sampling was performed in 2013. These analyses were carried out only with *V. crassostreae*, due to the absence of *V. tasmaniensis* detection in 2014 samples. Results showed no significant correlation between *V. crassostreae* and microzooplankton species (Table 2).

### *Vibrio tasmaniensis* Association to the Cnidarian Hydrozoan *Obelia* sp.

Next, we tested whether plankton taxa whose abundance correlated with *Vibrio* spp. abundance were PCR-positive for pathogenic vibrios. We isolated individuals from *Obelia* sp., *Centropages* sp., and *O. dioica* from PCR-positive samples and performed PCR detection on the isolated specimens. As a negative PCR control (no PCR detection expected), we used annelid specimens isolated from our PCR-positive field samples, as their temporal dynamics did not show any positive correlation with any *Vibrio* species potentially pathogenic to oysters. Samples were analyzed with *16S rRNA, ompU, R5-2*, and *dnaJ* primers. All isolated individuals were positive for *16S rRNA*, indicating that *Vibrio* species were associated with a broad number of plankton species (Supplementary Table S2). We could not confirm the interactions of *V. crassostreae* with *Centropages* sp. and *Oikopleura* sp., probably due to the limited number of individuals that were isolated. However, in agreement with our statistical analyses from 2013, PCR conducted on *Obelia* sp. was positive for *V. tasmaniensis* but not for *V. crassostreae* nor *V. aestuarianus* (Supplementary Table S2).

### Sediment as a Possible Winter Reservoir of *V. tasmaniensis* and *V. crassostreae* in Oyster Farms

**FIGURE 6:**
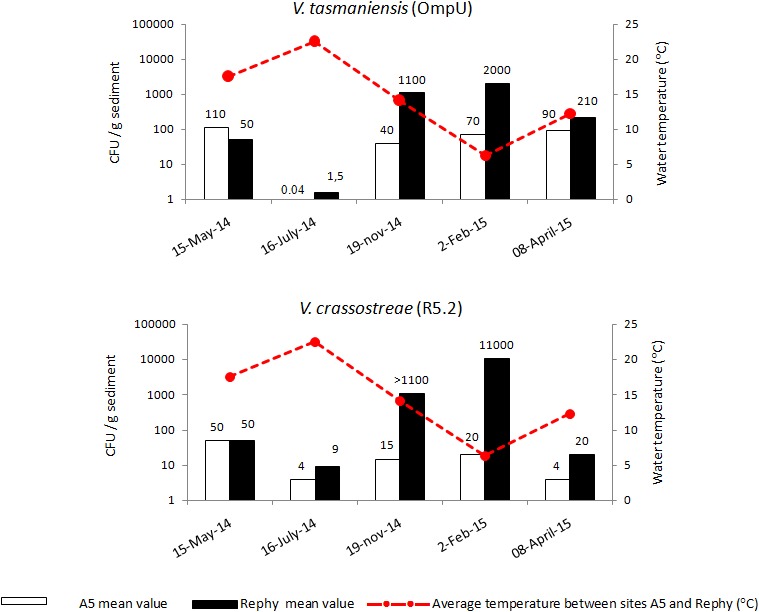
Seasonal abundance of vibrios in the sediment. Mean concentration of *Vibrio tasmaniensis* and *Vibrio crassostreae* (MPN CFU/g) in the surface sediment collected outside the oyster culture area (A5, *white bar*) and inside an oyster farm (Rephy, *black bars*). Mean subsurface seawater temperature (°C) is represented by a red line.

To determine if compartments other than the water column, such as the sediment, could serve as a reservoir for *Vibrio* species potentially pathogenic to oysters, we collected sediment at the main sites surveyed (Rephy and A5) during all four seasons (five samplings from May 2014 to April 2015). *V. tasmaniensis* (0.04–110 CFU MPN/g) and *V. crassostreae* (4–50 CFU MPN/g) were scarce in the sediment outside oyster farms (A5) (Figure 6). In contrast, the sediment from oyster farms (Rephy) contained remarkably higher levels of both pathogenic populations in autumn (1.1 × 10^3^ CFU MPN/g) and winter (2 × 10^3^ and 1.1 × 10^4^ CFU MPN/g for *V. tasmaniensis* and *V. crassostreae*, respectively) (Figure 6). Both *V. tasmaniensis* and *V. crassostreae* showed a major decline in the sediment in summer, irrespective of station surveyed. Statistical analysis revealed a significant negative correlation between the abundance of *V. tasmaniensis* and temperature at the Rephy station (*r* = 0.915, *p* < 0.05). The same trend was observed for *V. crassostreae* at the Rephy station, but the correlation was not significant (*r* = 0.795, *p* > 0.05).

## Discussion

The present study reports the environmental dynamics of *Vibrio* species potentially associated with the Pacific oyster mortality syndrome in a Mediterranean lagoon highly exploited for oyster culture. By monitoring both total vibrios and potential pathogenic species over 2 years (2013–2014) characterized by mass mortality of oyster juveniles, we could identify important parameters influencing their ecology as well as potential environmental reservoirs. We could also investigate the effect of oyster culture on pathogen cycles.

Our 2-year survey revealed that *Vibrio* species were permanently present in the Thau Lagoon, both as free-living and plankton-associated bacteria in all water column fractions during 2013–2014. However, *Vibrio* species exhibited contrasting abundances over the 2 years, which were otherwise characterized by similar trends in temperature. Indeed, *Vibrio* cells were ∼100-fold more numerous in 2013 than in 2014. Overall, the annual dynamics of total *Vibrio* spp. did not correlate with temperature, thus supporting recent findings by Vezzulli et al. (2015) in another Mediterranean lagoon. Instead, phyto- and zooplankton levels, which were much higher in 2013 than in 2014, correlated with vibrio abundances, suggesting they could promote the increase of vibrios in the water column. Several studies have shown a close relationship between plankton community structure and vibrio abundance (Asplund et al., 2011; Main et al., 2015). Our results demonstrate that interactions between vibrios and plankton species were dependent not only on plankton abundance but also on the specific taxa present in the water column.

*Vibrio* species potentially pathogenic to oysters were abundant in the water column of the Thau Lagoon 1 month after major episodes of spat mortalities. Particularly, *V. tasmaniensis* and *V. crassostreae* from the Splendidus clade, which contributes a large proportion of pathogenic strains (Gay et al., 2004; Bruto et al., 2017), were detected at significant levels in the water column. The highest concentration of pathogenic *Vibrio* species reached up to 10^7^ cells L^-1^ of seawater, that is 15 times more than the concentrations reported for the entire Splendidus clade in the Goro Lagoon (Italy), which is not used for oyster farming (Vezzulli et al., 2015). It is tempting to speculate that pathogens are released by oysters themselves as a consequence of the disease, although other environmental reservoirs could also contribute to the pathogens’ cycle. Remarkably, no difference in abundance of each of these potentially pathogenic vibrios was observed between sampling sites located inside and outside oyster farms (1.5–2 km apart). This is consistent with previous data showing that the disease (measured in terms of oyster mortality) is easily and rapidly transmitted from site to site in the Thau Lagoon, in an epizootic process of local transmission (Pernet et al., 2014). Our data further indicate that pathogenic vibrios rapidly disperse both as free-living or plankton-associated populations not only from farm to farm but also outside oyster farming areas. Such dispersal is probably mediated by both the hydrodynamic connectivity of the Thau Lagoon (Lazure and Dumas, 2008; Fiandrino and Verney, 2010) and the vibrios’ own motility, which allows them to spread into favorable environments and/or escape from unfavorable ones (Zhu et al., 2013). Among the three pathogens surveyed, only *V. aestuarianus* was not detected over the 2 years, which suggests a low prevalence of this species in the Thau Lagoon.

The ecology of *Vibrio* species potentially pathogenic to oysters was found to be influenced by different environmental drivers. The abundance of *V. crassostreae* in the water column correlated with temperature, in agreement with previous studies showing that salinity and temperature correlated with vibrio abundance (Thompson et al., 2004; Turner et al., 2009; Wendling and Wegner, 2013). Interestingly, among the two potential pathogens present in the Thau Lagoon, only *V. crassostreae* was repeatedly observed both as free-living and plankton-associated in the warm months of 2013 and 2014 (from May to September). In contrast, *V. tasmaniensis* was found mainly in 2013, adopting a free-living mode of life in the warm months (May–August) while being plankton-associated throughout the year. Whereas temperature and salinity often explain more of the variation in *Vibrio* abundance than any other water parameter, Takemura et al. (2014) showed that such trends did not necessarily apply to *Vibrio* species in a natural environment. The switch between a free-living and plankton-associated mode of life observed for *V. tasmaniensis* suggests an important contribution of plankton composition to its dynamics in the water column. Our data suggest that the dynamics of *V. tasmaniensis* populations could depend substantially on interactions between *V. tasmaniensis* and the cnidarian *Obelia* sp. Thus, from our study, the dynamics of *V. crassostreae* in the Thau Lagoon appears driven by both temperature and plankton composition, whereas the dynamics of *V. tasmaniensis* appears determined only by the latter. This is consistent with previous reports showing that the interaction between *Vibrio* and plankton species was influenced by changes in plankton community structure and abundance (Main et al., 2015) and that plankton taxa were important factors explaining *Vibrio* abundance (Takemura et al., 2014).

Both pathogenic species of *Vibrio, V. crassostreae* and *V. tasmaniensis*, were abundant on a broad spectrum of planktonic hosts (>20 μm), including species of copepods, ciliates, cnidarians, tunicates, diatoms, and dinoflagellates. Although they probably do not directly infect these species, pathogenic vibrios can attach to their surface and behave as multi-host pathogens. These associations can provide a major boost for transmission. Indeed, oysters feed more easily on large particles than on free-living bacteria (Dupuy et al., 2000). Moreover, we observed that oyster farming had a strong negative impact on plankton abundance, most likely as a consequence of oyster filtration activity. Therefore, attachment of pathogenic *Vibrio* species to plankton taxa could substantially increase transmission rate to oysters, as proposed for the transmission of *Vibrio cholera*e to humans (Rawlings et al., 2007). Association with living organisms is also known to be one of the main survival strategies for *Vibrio* species, which allows them to persist in the pelagic environment (Dawson et al., 1981). From an evolutionary point of view, this suggests that *Vibrio* species pathogenic to oysters have evolved mechanisms for plankton attachment that not only favor persistence in the environment, but also foster their efficient transmission and infectivity. Whether the same adhesins are involved at different stages or in different environments is of major importance for understanding pathogen emergence.

Notably, the sediment was identified here as a potential winter reservoir of *V. tasmaniensis* and *V. crassostreae* in oyster farms of the Thau Lagoon. Indeed, a marked increase in *V. tasmaniensis* and *V. crassostreae* abundance was observed in the sediment below oyster tables in autumn and winter. Our field data show that high temperatures had a negative effect on *V. tasmaniensis* abundance both in the sediment and in the water column. This is in agreement with Vezzulli et al. (2015), who observed (i) high amounts of *Vibrio* cells in the sediment of the Goro Lagoon during winter, and (ii) a better persistence of *V. tasmaniensis* LGP32 at 5°C than at 25°C. As *Vibrio* abundance remained low outside oyster tables in the Thau Lagoon over the entire period surveyed, oyster farming appears to actively enrich the sediment with pathogenic vibrios. This is probably due to the mass mortalities observed in spring and autumn and the subsequent deposition of bacteria in the sediment of this shallow area (<4 m). Thus, during winter, the sediment under the oyster tables could contribute to the persistence of oyster pathogens in the Thau Lagoon. We hypothesize that vibrios find there a source of nutrients and/or establish biotic interactions with benthic organisms that favor their viability and growth. It is likely that the high abundance of pathogens in the surface sediment seeds the water column upon resuspension, as demonstrated for the neurotoxic cyst-forming dinoflagellate *Alexandrium catenella* (Genovesi et al., 2013) in the Thau Lagoon. This finding deserves particular attention in terms of oyster farming management. Indeed, common practices such as increasing oyster biomass to compensate for mortalities may have significant impact by seeding the sediment of oyster farms with pathogenic vibrios, thereby nurturing pathogen cycles in this highly exploited marine ecosystem.

In summary, the present study offers new field data to identify epidemiological patterns linked to *Vibrio* community dynamics, especially species pathogenic for the Pacific oyster *C. gigas*. Our results indicate that oyster farming contributes to the pathogens’ life cycle, particularly through oyster farm sediments, which were found to contain abundant pathogenic vibrios during the winter season. Accordingly, sediments could constitute an important seeding reservoir. Although pathogens were sporadically detected in the water column in winter, probably upon resuspension of sediment, they thrived mainly when temperature increased, after oyster mortality events started. The differing abundance and composition of plankton and vibrios over the 2 years surveyed revealed the complexity of pathogen ecology in this Mediterranean ecosystem. As there is considerable economic interest in identifying which factors determine the ecology of pathogenic vibrios, the conclusions from the present work should be reinforced by large-scale monitoring of the Thau Lagoon. This would include monitoring pathogens and plankton composition over more years and throughout the lagoon, taking into account the hydrodynamic properties of the ecosystem. As pathogenicity of vibrios does not depend on single molecular determinants, which can additionally be shared by distinct pathogenic populations as recently illustrated by Bruto et al. (2018), specific tools to accurately monitor pathogens are still missing. Therefore, isolation, genotyping, and phenotyping of *Vibrio* strains associated with the different ecological compartments in the marine ecosystem of interest will have to be considered in the future.

## Author Contributions

DD-G, J-LR, DB, and AC conceived and designed the experiments. CL-J, J-LR, PH, AC, CC, DB, CR, EA, and ML performed the experiments. J-LR, CL-J, M-AT, AC, and DB analyzed the data. CL-J, J-LR, DB, AC, and DD-G wrote the paper.

## Conflict of Interest Statement

The authors declare that the research was conducted in the absence of any commercial or financial relationships that could be construed as a potential conflict of interest.

## Footnotes

1 http://wwz.ifremer.fr/observatoire_conchylicole

2 http://wwz.ifremer.fr/lerpc/Activites-et-Missions/Surveillance/REPHY
