# Supplementary material for: Oyster Farming, Temperature, and Plankton Influence the Dynamics of Pathogenic Vibrios in the Thau Lagoon
Source: Front Microbiol. 2018 Oct 24;9:2530. doi: 10.3389/fmicb.2018.02530 (PMC6207591; doi:10.3389/fmicb.2018.02530)
Supplement: Supplementary file 1 [file Table_1.PDF]

Supplementary Table S1. Specificity of primer pairs used for the detection of vibrios. DNA extracted from a diversity of *Vibrio* strains was subjected to PCR using primers designed on *ompU, dnaJ* and *R5-2* sequences specific for the oyster pathogen *V. tasmaniensis*, *V. aestuarianus and V. crassostreae,* respectively. Primers amplifying 16S rRNA sequences form all vibrios were also used. Positive and negative amplifications are indicated by **+** and **–** respectively. NT not tested.

Species	Strain	*Virulence in oysters*	*Reference*	*ompU*	*dnaJ*	*R5-2*	*16S rRNA*
**Splendidus clade**							
V. tasmaniensis	LGP32	Vir (+)	Gay M. et al. 2004	+	-	-	+
	J5-9	Vir (+)	Lemire et al. 2015	+	-	-	+
	J5-13	Vir (+)	Lemire et al. 2015	+	-	-	+
	J0-13	Vir (+)	Lemire et al. 2015	+	-	-	+
	Med222	NT	Ifremer Institute, France	+	-	-	+
	LMG20012T	Vir (-)	Ifremer Institute, France	-	-	-	+
V. splendidus	J5-1	Vir (+)	Lemire et al. 2015	-	-	-	+
	J2-23	Vir (-)	Lemire et al. 2015	-	-	-	+
V. crassostreae	J5-4	Vir (+)	Lemire et al. 2015	-	-	+	+
	J2-9	Vir (+)	Lemire et al. 2015	-	-	+	+
V. cyclitrophicus	J2-5	Vir (+)	Lemire et al. 2015	-	-	-	+
V. sp.	J2-8	Vir (-)	Lemire et al. 2015	-	-	-	+
**Other clades**							
V. tapetis	CECT4600	NT	Paillard and Maes, 1990	-	-	-	+
V. fischeri	ES114	NT	Mandel *et al*, 2008	-	-	-	+
V. aestuarianus	02_041	Vir (+)	Ifremer Institute, France	-	+	-	+
V. tubiashii	CRL10	NT	Ifremer Institute, France	-	-	-	+
V. penaeicidae	AM101	NT	Ifremer Institute, France	-	-	-	+
V. nigripulchritudo	SFn1	NT	Ifremer Institute, France	-	-	-	+
V. metshnikovii	NCTC8483	NT	Pasteur Institute, France	-	-	-	+
V. harveyi	ATCC14126	NT	Pasteur Institute, France	-	-	-	+
V. anguillarum	ATCC19264	NT	Pasteur Institute, France	-	-	-	+
