# Supplementary material for: Oyster Farming, Temperature, and Plankton Influence the Dynamics of Pathogenic Vibrios in the Thau Lagoon
Source: Front Microbiol. 2018 Oct 24;9:2530. doi: 10.3389/fmicb.2018.02530 (PMC6207591; doi:10.3389/fmicb.2018.02530)
Supplement: Supplementary file 2 [file Table_2.PDF]

Supplementary Table S2. **PCR-detection of vibrios on plankton specimen isolated from vibrio-positive water column samples.** DNA extracted from specimens of plankton species (15 to 76 individuals) collected in *situ,* was subjected to PCR using primers designed on *ompU, dnaJ* and *R5-2* sequences specific for the oyster pathogen *V. tasmaniensis*, *V. aestuarianus and V. crassostreae,* respectively. Primers amplifying 16S rRNA sequences form all vibrios were also used. Sampling date, sampling stations and positive correlations are reported in the table. Positive and negative amplifications are indicated by **+** and **–** respectively.

Species	*Correlation*	*Sampling station*	*Sampling date*	*Number of individuals*	*PCR detection*			
					ompU	*dnaJ*	*R5-2*	*16S rRNA*
Obelia sp.	*V. tasmaniensis* LGP32, r = 0.697	A5	june 2013	76	+	-	-	+
Oikopleura sp.	*V. crassostreae*, r = 0.482	A5	june 2013	15	-	-	-	+
		REPHY	june 2013	48	-	-	-	+
Centropages sp.	*V. crassostreae*, r = 0.733	A5	june 2013	47	-	-	-	+
Annelides sp.	no correlation	A5	june 2013	32	-	-	-	+
