# Supplementary figures and images for: Oyster Farming, Temperature, and Plankton Influence the Dynamics of Pathogenic Vibrios in the Thau Lagoon
Source: Front Microbiol. 2018 Oct 24;9:2530. doi: 10.3389/fmicb.2018.02530 (PMC6207591; doi:10.3389/fmicb.2018.02530)

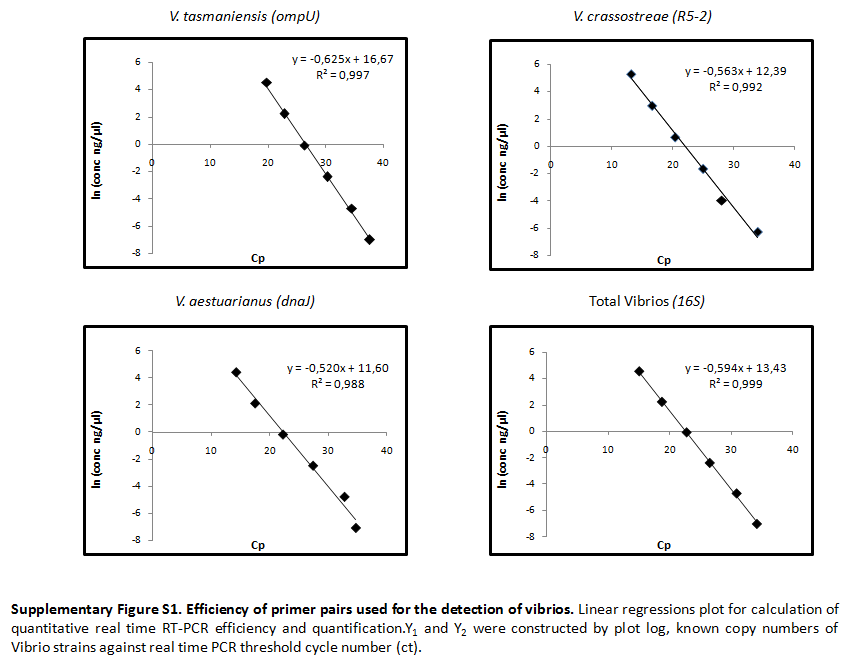

Supplement: Supplementary file 3 [file Image_1.TIF]

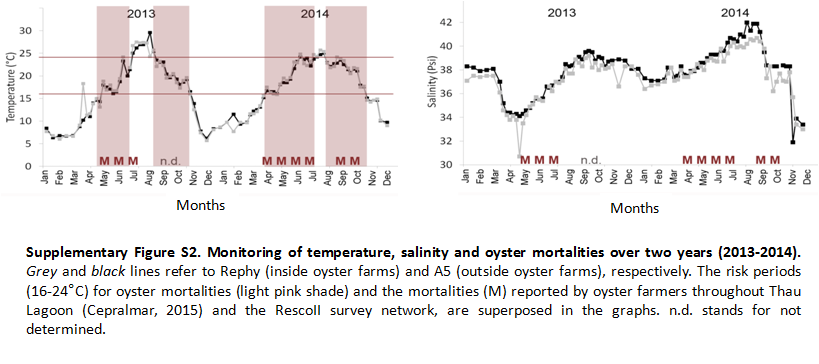

Supplement: Supplementary file 4 [file Image_2.TIF]

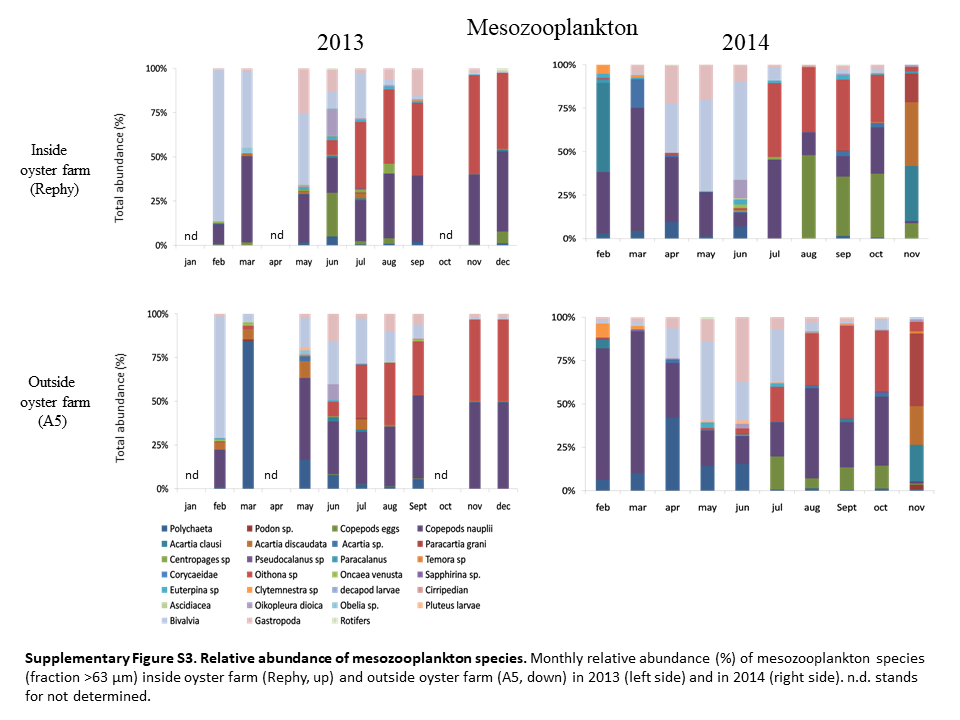

Supplement: Supplementary file 5 [file Image_3.tif]

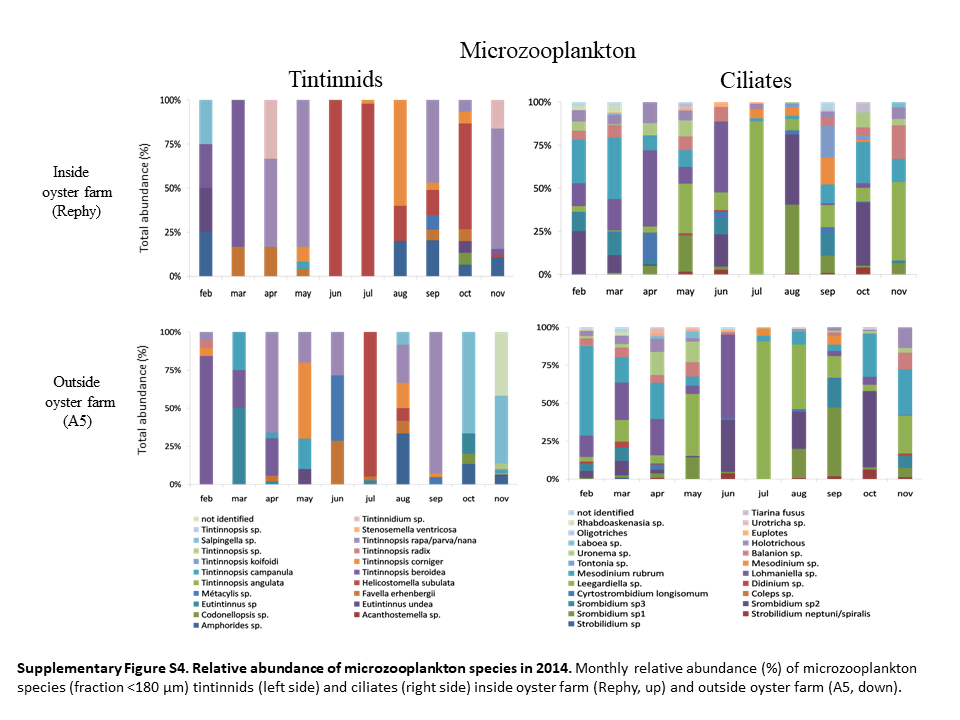

Supplement: Supplementary file 6 [file Image_4.tif]

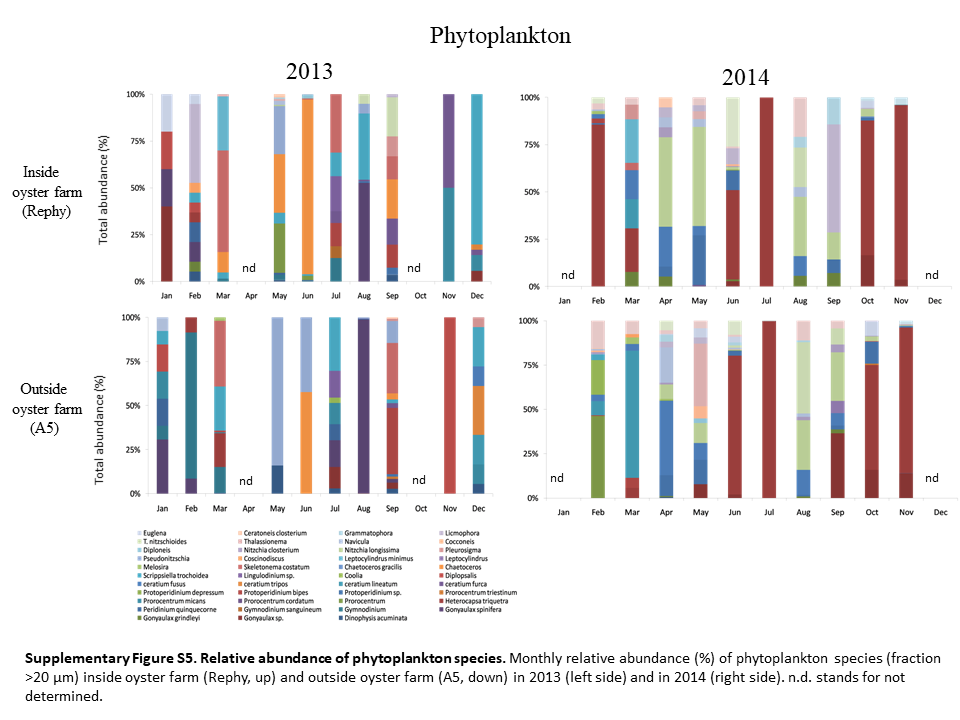

Supplement: Supplementary file 7 [file Image_5.tif]
